# Control of muscle satellite cell function by specific exercise‐induced cytokines and their applications in muscle maintenance

**DOI:** 10.1002/jcsm.13440

**Published:** 2024-02-20

**Authors:** Qian Guo, Qing Luo, Guanbin Song

**Affiliations:** ^1^ Key Laboratory of Biorheological Science and Technology, Ministry of Education, College of Bioengineering Chongqing University Chongqing China

**Keywords:** Cytokines, Exercise, Homeostasis, Muscle satellite cells, Skeletal muscle

## Abstract

Exercise is recognized to play an observable role in improving human health, especially in promoting muscle hypertrophy and intervening in muscle mass loss‐related diseases, including sarcopenia. Recent rapid advances have demonstrated that exercise induces the release of abundant cytokines from several tissues (e.g., liver, muscle, and adipose tissue), and multiple cytokines improve the functions or expand the numbers of adult stem cells, providing candidate cytokines for alleviating a wide range of diseases. Muscle satellite cells (SCs) are a population of muscle stem cells that are mitotically quiescent but exit from the dormancy state to become activated in response to physical stimuli, after which SCs undergo asymmetric divisions to generate new SCs (stem cell pool maintenance) and commit to later differentiation into myocytes (skeletal muscle replenishment). SCs are essential for the postnatal growth, maintenance, and regeneration of skeletal muscle. Emerging evidence reveals that exercise regulates muscle function largely via the exercise‐induced cytokines that govern SC potential, but this phenomenon is complicated and confusing. This review provides a comprehensive integrative overview of the identified exercise‐induced cytokines and the roles of these cytokines in SC function, providing a more complete picture regarding the mechanism of SC homeostasis and rejuvenation therapies for skeletal muscle.

## Introduction

Exercise is a structured and repetitive physical activity that includes endurance (aerobic) training (e.g., walking, jogging, and cycling) and resistance (anaerobic) training (e.g., barbell bench presses, barbell overhead squats, and dumbbell curls).^1^, ^2^ Exercise has been widely recognized as an effective approach for enhancing muscle performance and relieving multiple chronic diseases, including diabetes, cardiovascular, sarcopenia, respiratory, and arthritic diseases (Data S1).^2^ Skeletal muscle loss is one of the major health hazards because a progressive decline in muscle mass and strength occurs with age and is defined as sarcopenia.^3^ Muscle satellite cells (SCs) are a population of muscle stem cells that are located between the basal lamina and the plasma membrane of muscle fibres.^4^, ^5^ SCs are essential for muscle growth and regeneration by generating numerous proliferating myoblasts that subsequently differentiate into myocytes.^5^, ^6^ The depletion of SCs blunts hypertrophy induced by exercise (progressive weighted wheel running),^7^ suggesting that SCs contribute to exercise‐induced hypertrophy. Over the past few years, considerable efforts have been made to elucidate the basic and vital question: how does exercise regulate SCs to enhance muscle performance?

Due to the beneficial effects of exercise on human health, screening exercise mimetics shows promise for clinical application and has attracted widespread attention. Several exercise mimetics, including the AMP‐activated protein kinase (AMPK) agonist, peroxisome proliferator‐activated receptors (PPAR) δ agonist, and irisin, have been demonstrated to greatly enhance muscle performance.^8^, ^9^ For instance, exercise activates multiple serine/threonine kinases, including AMPK, in skeletal muscle.^S2^ AMPK regulates the activation of PPARs via the PPARγ coactivator 1α (PGC‐1α).^S3^ Both AICAR (an AMPK agonist) and GW1516 (a PPARδ agonist) substantially enhance the running endurance of mice.^8^ Moreover, the combination of exercise and the AMPK/PPARδ agonist significantly improved the muscle performance of mdx mice.^S4^ Notably, AICAR treatment improved the muscle performance in obese mice, but AMPKα1 deficiency in SCs abolished this phenomenon, demonstrating that the functions of AMPK agonists largely depend on SCs.^S5^

Emerging evidence indicates that the effect of exercise on tissues is largely mediated by circulating molecules induced by exercise, including myokines, hepatokines, osteokines, immune cytokines, and adipokines.^10^, ^11^ Several exercise‐induced cytokines, such as irisin,^12^ erythropoietin,^13^ and follistatin,^14^ ^,S6^ promote the activation of SCs and substantially enhance muscle performance. Some exercise‐induced cytokines, such as Igfbp7 and myostatin, preserve SC quiescence or inhibit SC self‐renewal.^15^, ^16^ The cytokines induced by exercise constitute a complex and diverse regulatory network for SCs that ultimately govern skeletal muscle function.^17^ This review aims to summarize and discuss the effect of exercise‐induced cytokines on SCs, providing in‐depth insight into SC homeostasis and rejuvenation therapies for skeletal muscle.

## Exercise regulates satellite cell fate decisions

### Satellite cell fate decisions

SCs are a heterogeneous population of adult muscle stem cells that reside mainly in a niche below the basal lamina and adjacent to the plasma membrane of myofibres.^18^ SCs are identified by the expression of the transcription factor Pax7.^19^ Myogenic regulatory factors (e.g., MyoD, Myf5, myogenin, and Mrf4) play critical roles in establishing myogenic cell identity and subsequent differentiation and formation of muscle fibres.^5^ The absence of Pax7^+^ cells resulted in a myofibre size proportional to the reduction in myonuclear cell number.^20^ SCs are normally in a dormant state with transient cell cycle inhibition (quiescence). In response to muscle injury or stress, quiescent SCs are activated and undergo several cycles of proliferation, and proliferative SCs either differentiate into myoblasts or return to quiescence to reconstitute the SC pool.^5^

A balanced transition between quiescence and activation is essential for the long‐term maintenance of SCs and muscle homeostasis.^21^ This process is governed by intrinsic factors (e.g., cell cycle regulators, transcription factors, and epigenetic factors) and niche factors (e.g., niche cells, blood vessels, the extracellular matrix, and cytokines).^5^ Nevertheless, it is becoming more widely acknowledged that niche factors govern the fate decisions of SCs via intracellular signalling pathways. SC transplantation experiments have shown that SCs adopt different fates when introduced to different environments,^22^ emphasizing the critical roles of niche factors in SC fate decisions. SCs are activated by various physiologic and pathologic stimuli, such as chronic inflammation, injury, exercise, and sarcopenia.^6^ In a classic model of muscle injury‐induced SC activation, immune cells rapidly increase in number in skeletal muscle.^23^ Neutrophils first reach the injury site and recruit and initiate the polarization of macrophages,^23^ while macrophages promote the activation and proliferation of SCs through release factors, such as insulin‐like growth factor 1, ADAMTS1 (which inhibits Notch signalling in SCs), and glutamine (which activates mTOR signalling in SCs).^S7‐S9^ Moreover, the extracellular ECM, nerve fibres, fibro/adipogenic progenitors, and endothelial cells also regulate the transition between quiescence and activation of SCs.^5^

### Exercise activates satellite cells and increases their pool

SCs have been identified as mechanically sensitive cells and physical activity has been demonstrated to be an essential factor for SC maintenance.^24^, ^25^ ^,S10^ It has been reported that primary cilia in SCs function as mechanical sensors for exercise, and depletion of ARL3 impairs cilia function and inhibits hedgehog signalling, which initiates myogenic programmes.^26^ Abundant evidence has demonstrated that exercise induces the activation of SCs via multiple pathways.^6^, ^27^ ^,S11^ Notably, several endocrine factors are critical mediators of exercise‐induced SC activation.^27^ For instance, nitric oxide levels are elevated by exercise,^28^ which has been reported to activate SCs via the activation of guanylate cases and the generation of cyclic guanosine monophosphate.^S12^ Moreover, the exercise‐induced cytokines we described in this review both substantially activate SCs (e.g., hepatocyte growth factor, irisin, and interleukin 6). In addition to exercise‐induced activation, exercise has been reported to enhance the self‐renewal of SCs via various pathways, including the inhibition of mitochondrial oxygen consumption^29^ and Akt–mTOR signalling.^15^ The excessive activation of SCs often causes exhaustion of the SC pool.^30^ We speculate that this adaptation of SCs under exercise preserves activated SCs without the expense of exhaustion.

It has been widely confirmed that exercise (e.g., running, swimming, and resistance training) increases SC abundance in humans and animals.^6^, ^31^ ^,S13^ For instance, voluntary wheel running for 14 days increased Pax7^+^BrdU^+^ activated SCs in mice.^S14^ After 14 weeks of lower body endurance and upper body resistance training the SC pool in the elderly increased by ~38%, but the myonuclear number and domain showed no significant change, suggesting that SCs are more sensitive to physical activity.^S15^ Conversely, unloading by hindlimb suspension in rats resulted in a 48–57% reduction in quiescent SCs.^S10^ Fourteen days of bed rest resulted in a reduction in SC content (~40% loss) and muscle atrophy in middle‐aged adults.^24^ The number of SCs in the rat central region muscle substantially decreased after 16 days of unloading; however, 16 days of reloading increased the SC content.^25^ These findings reveal that the maintenance of SCs requires physical activity.

### Satellite cells contribute to exercise‐induced muscle adaptation

It is widely recognized that regular exercise alters the phenotype of skeletal muscle, involving changes in metabolic programmes and structural proteins within myofibres.^8^, ^32^ ^,S16^ At the molecular level, skeletal muscle shows increased expression of genes associated with the slow‐twitch contractile apparatus, mitochondrial respiration, fatty acid oxidation, and protein synthesis.^8^ ^,S17,S18^ These adaptations induce muscle hypertrophy, enhancing muscle strength, and facilitating muscle regeneration.^32^

Emerging evidence demonstrates that SCs substantially contribute to exercise‐induced muscle adaptation. Exercise improved skeletal muscle regeneration,^15^, ^33^ ^,S19^ which has been partially attributed to the enhanced self‐renewal of SCs.^15^, ^29^ It is widely recognized that prolonged exercise promotes muscle hypertrophy (increased muscle mass and fibre size) in humans.^27^ Muscle hypertrophy is another primary adaptation to exercise; however, whether SCs are essential for exercise‐induced muscle hypertrophy remains controversial. After 2 weeks of overload, the mice with PAX7^+^ SC depletion exhibited the same increase in muscle mass as the hypertrophy observed in the control group.^S20^ Because there are discrepancies between muscle mass and fibre cross‐sectional area (CSA) in the plantaris, another study utilized fibre CSA as a proxy for fibre hypertrophy and demonstrated that SC depletion prevents fibre hypertrophy in skeletal muscle.^34^ Further research reported that PAX7^+^ SC depletion prevented overload‐induced hypertrophy (shown by fibre CSA) in young mice (2 month old) but not in mature mice (4 months old).^S21^ Depletion of Pax7^+^ SCs in mice resulted in blunted muscle growth in response to progressive weighted wheel running compared with that in wild‐type mice.^7^ Moreover, the absence of SCs largely alters myonuclear transcriptional coordination in response to exercise, revealing crosstalk between SCs and myocytes.^7^ ^,S22^

An increased muscle fibre size is commonly associated with an increased number of myonuclei, while the myonuclear domain theory of muscle growth notes that the addition of new myonuclei comes from a new source donated from SCs.^S11^ Thus, the response of SCs to exercise is thought to occur for the following purposes: (1) SC activation under exercise benefits myonuclear addition to maintain the myonuclear domain and (2) SC self‐renewal under exercise benefits SC maintenance and muscle regeneration. Taken together, the current evidence suggests that exercise‐induced muscle hypertrophy may be initiated and sustained for a period in the absence of SCs, but exercise‐induced muscle change ultimately requires SC to achieve full adaptive potential. At present, how exercise regulates SC function is unclear because exercise induces systemic changes.^6^, ^27^ ^,S11^ Thus, elucidating how exercise regulates SCs will improve the understanding of SC homeostasis and provide intervention targets.

### Specific exercise‐induced cytokines govern satellite cell function

#### Identification of exercise‐induced cytokines

Cytokines are defined as small proteins with a wide range of biological activities and are released by various organs and tissues, mainly skeletal muscle, adipose tissue, liver, bone, and brain.^35^ Cytokines that are mainly released from hepatocytes, immune cells, or adipocytes are defined as hepatokines, immune cytokines, and adipokines, respectively. Skeletal muscle accounts for 30–50% of the total body mass and is a major secretory organ that releases multiple cytokines and other peptides; these molecules are classified as myokines.^10^ A proximity labelling strategy for myocyte secretome profiling in mice uncovered hundreds of proteins.^S23^ Based on an exercise mouse model (1‐week treadmill running for 60 min/day at a speed of 20 m/min) and a biomedical secretome profiling methodology, has identified >200 exercise training‐regulated protein pairs (21‐cell‐type, 10‐tissue), uncovering a complex map of exercise‐regulated cytokines.^36^

Emerging evidence has demonstrated that exercise‐induced cytokines are critical mediators of the beneficial effects of exercise.^12^, ^37^, ^38^, ^39^, ^40^ Among exercise‐induced proteins, carboxylesterase enzymes secreted from the liver exhibit anti‐obesity effects and enhance running endurance in mice.^36^ Irisin, a cleaved and circulating form of the exercise‐induced protein FNDC5, promotes the browning of white adipose tissue, induces muscle hypertrophy by activating SCs, and is sufficient to confer the benefits of exercise on cognitive function.^12^, ^37^ It has also been revealed that exercise induces an increase in clusterin in the blood, which substantially dampens brain inflammation.^39^ The anti‐tumour effect of exercise is also partially attributed to exercise‐induced cytokines because metabolites released from skeletal muscle following exercise enhance the effector profile of CD8^+^ T cells.^S24^ Various cytokines, such as irisin, myostatin, insulin‐like growth factor‐1 (IGF‐1), hepatocyte growth factor (HGF), basic fibroblast growth factor (bFGF), and interleukin‐6 (IL‐6), have been demonstrated to control SC functions.^5^, ^41^ Increasing evidence reveals that exercise‐induced cytokines play vital roles in controlling SC activation, proliferation, differentiation, and self‐renewal.^2^, ^11^, ^42^ Here, we discuss the sources of exercise‐induced cytokines, their functions in SCs, and potential applications in muscle maintenance (Table 1).

**Table 1:** Summary of exercise‐induced cytokines and their impacts on SCs

Cytokine	Main source	Impact on SCs	Therapeutic potential
Myostatin	Myocytes	Preserving proliferation^S28^; inhibiting proliferation and differentiation ^S29^	Unloading‐related muscle loss^43^, ^44^
Irisin	Myocytes	Promoting activation and differentiation^12^	Muscle atrophy^12^, ^45^
IL‐6	Myocytes, blood cells	Promoting activation^46^, ^47^	Muscle atrophy^47^
Igfbp7	Myocytes, SCs	Promoting self‐renewal^15^	Muscle atrophy and regeneration^15^
G‐CSF	Myocytes	Promoting asymmetric division^48^	Age‐related sarcopenia^48^
HGF	Myocytes, fibroblasts	Promoting activation,^49^, ^50^ inhibiting differentiation^51^	Muscle atrophy^52^
CXCL10	Macrophages	Promoting proliferation^53^	Age‐related sarcopenia^53^
Nampt	Macrophages	Promoting proliferation^54^	Muscle regeneration^54^
CXCL12	Blood cells, myocytes	Promoting differentiation^55^	
Follistatin	Hepatocytes	Promoting activation and proliferation^S6^	Muscle atrophy^S6^
Adiponectin	Adipocytes	Inducing quiescence exit^56^	Muscle regeneration^56^
Leptin	Adipocytes	Inducing quiescence exit^57^	Muscle regeneration^57^, ^58^
BDNF	Brain, myocytes	Preserving proliferation^59^, ^60^	Muscle regeneration^59^, ^60^
EPO	Kidney, liver, myocytes	Promoting proliferation,^13^ inhibiting apoptosis^61^	Muscle atrophy^13^

#### Myokines‐myostatin

Myostatin also referred to as growth differentiation factor 8 (GDF‐8), belongs to the transforming growth factor β (TGF‐b) superfamily. Myostatin is a myokine involved in the maintenance of metabolic homeostasis and the regulation of adipose tissue function.^10^, ^62^ Acute exercise increases the concentration of myostatin in serum in humans^63^ ^,S25^; however, another study showed a decrease in the concentration of myostatin in serum in healthy young men 24 h after resistance,^64^ which may be attributed to the different time points of blood sampling. Mutation or knockout of myostatin results in muscle hypertrophy in humans and mice.^65^, ^66^ ^,S26^ Myostatin binds to receptors on the cell membrane to induce atrophy signalling by activating Smad‐Atrogin1/MuRF1 signalling and blocking the transcription of myogenic genes (e.g., Pax7, MyoD, MyoG, and MyHC).^S27^ At present, the role of myostatin in SCs is not yet well understood. It has been reported that the knockdown of myostatin reduces the proliferation of equine SCs,^S28^ but other research has shown that the knockdown of myostatin promotes the proliferation and myogenic differentiation of bovine SCs.^S29^ At the molecular level, myostatin interacts with the extracellular matrix‐related protein type I collagen α 1 (COL1A1) to inhibit adhesion, the PI3K‐AKT pathway, and the ribosome pathway in bovine SCs.^S29^ Moreover, myostatin has been identified as a major effector of SC unloading. Microgravity induces myostatin expression in SCs, while the use of anti‐myostatin antibodies preserves cell survival and differentiation of SCs,^43^, ^44^ suggesting that myostatin is an exercise‐sensitive cytokine and an intervention target for unloading‐related muscle loss.

#### Myokines‐irisin

Irisin is a 112‐amino‐acid‐long hormone generated by the cleavage of fibronectin type III domain containing 5 (FNDC5).^S30^ Irisin is secreted mainly by skeletal muscle and is defined as a myokine. It has been found that adipose tissue and the liver also secrete a few irisin.^S30‐S32^ It has been well recognized that irisin is induced by exercise in humans and mice.^67^ ^,S32,S33^ Mouse exposure to swimming or free wheel running, and human exposure to 10 weeks of aerobic training induced the secretion of irisin from skeletal muscle into blood, which has been demonstrated to increase energy expenditure by stimulating the ‘browning’ of white adipose tissue.^S34,S35^ In addition to the potential use of irisin as a therapeutic agent against obesity or type 2 diabetes, irisin also functions as a promyogenic factor. The serum irisin concentration was positively related to handgrip strength (*P* = 0.03) and low fall risk (*P* = 0.02) in 138 participants.^S36^ The serum irisin concentration has been identified as a predictive factor.^S37^ Treatment with irisin induced myogenesis in human primary myoblasts *in vitro*, while injection of irisin substantially promoted muscle hypertrophy and regeneration in mice, with enhanced grip strength.^12^ At the molecular level, irisin promoted the activation of SCs and protein synthesis, in association with increased phosphorylation of ERK1/2 and AKT.^12^ Moreover, irisin rescues muscle atrophy induced by denervation or unloading,^12^, ^45^ suggesting that irisin may be a possible therapy for counteracting muscle loss. At present, the effect of irisin on human skeletal muscle and the exact mechanism through which irisin activates SCs require further investigation.

#### Myokines‐interleukin 4/6

Interleukin (IL) is a class of cytokines that were originally found to be released by white blood cells, and more than 40 ILs have been identified, named IL‐1 to IL‐40.^68^ Recent evidence suggests that skeletal muscle is also a producer of severe ILs, including IL‐4, IL‐6, IL‐7, IL‐8, IL13, and IL‐15.^10^ ^,S38^ The expression of IL‐4 and IL‐13 in skeletal muscle is elevated after strength training.^S38^ IL‐4 administration to C26 colon carcinoma‐bearing mice rescues muscle mass, which is associated with the reestablished number and function of SCs.^S39^ IL‐4 has been demonstrated to directly stimulate myocyte differentiation of C2C12 cells (a mouse myoblast cell line).^S39^ Muscle cells are the dominant source of IL‐6, and exercise can induce a 100‐fold increase in the plasma of IL‐6 concentration; this increase is attributed to cytoplasmic Ca^2+^ and ionic enzymes in exercised skeletal muscle.^10^, ^69^ ^,S40,S41^ IL‐6 infusion led to muscle atrophy, as shown by a loss of myofibrillar protein in rats.^70^ However, additional evidence has shown that IL‐6 promotes the activation of SCs, while the loss of IL‐6 abolishes SC‐mediated muscle hypertrophy.^46^, ^47^ IL‐6 binds to the cell membrane IL‐6 receptor GP130 and induces the activation of JAK/STAT/cyclin D1 and MAPK signalling.^47^, ^71^ Loss of IL‐6 attenuates muscle hypertrophy induced by overload,^46^ and a recent theory suggested that IL‐6 acts as an energy allocator in muscle by regulating lipolysis, muscular energy uptake, and immune function.^S42^ In addition to IL‐6, exercise also induces the release of **the** myokines IL‐8, IL‐10, IL‐15, and IL‐16^41^, ^42^ ^,S43^; however, the roles of these ILs in SCs under exercise conditions remain unknown.

#### Myokines‐hepatocyte growth factor

HGF was originally identified as a mitogenic protein for *in vitro* culture of hepatocytes.^72^ HGF belongs to the group of plasminogen‐related growth factors/cytokines and is expressed mainly in fibroblasts, myocytes, and myoblasts.^49^, ^73^ HGF is a pleiotropic cytokine that supports the morphogenesis, regeneration, and survival of various cells.^73^ It has been demonstrated that supine exercise induces HGF production in patients with acute myocardial infarction.^S44^ For aortic valve stenosis patients and healthy persons, one‐hour peak bicycle exercise increased the blood levels of HGF.^S45^ The HGF protein was increased in the serum of humans at 4 h after muscle 300 lengthening contractions,^S46^ suggesting that HGF is an exercise‐induced cytokine. Moreover, stretched skeletal muscle induces HGF production in a nitric oxide‐dependent manner.^74^ The c‐met proto‐oncogene product of transmembrane receptor tyrosine kinase is the receptor for HGF.^73^, ^75^ The binding of HGF to Met induces the phosphorylation of multiple tyrosine residues within the cytoplasmic region, which further recruits intracellular signalling molecules.^73^ HGF promotes the activation of quiescent SCs *in vitro*, which is associated with increased expression of cyclin‐D1 and proliferating cell nuclear antigen.^76^ Notably, the administration of HGF significantly increased the number of Ki67^+^ cells of SCs in a mouse muscle atrophy model.^52^ HGF is present in an inert form in skeletal muscle and is activated by extracellular proteases to drive SC activation and proliferation upon muscle injury.^77^ The SCs of mice fed a high‐fat diet exhibited impaired activation and delayed regeneration, possibly because of the reduced HGF in skeletal muscle.^78^ However, the administration of HGF into muscle at the time of injury increased the number of myoblasts but inhibited muscle regeneration, which was attributed to the inhibitory effect of HGF on SC differentiation.^50^ HGF reportedly impedes the myogenic activity of SCs by inhibiting the activity of basic helix–loop–helix/E protein heterodimers, suggesting a dual role in SC myogenesis.^51^

#### Immune cytokines

Immune cells are also important mediators of the effect of exercise on muscle tissue. Exercise induces the release of the myokine IL‐6, which increases Treg abundance in skeletal muscle, while the depletion of Treg cells abolishes muscle‐specific gene expression that is required for the response to exercise (e.g., Hk2, Ckmt2, and Cpt1b).^79^ Two weeks of eccentric exercise (16 m/min, 90 min/day) dynamically altered the macrophage phenotype in rat skeletal muscle.^80^ Twelve weeks of endurance exercise training (cycle ergometer) increased the number of M2 macrophages in human skeletal muscle, which was positively associated with qfibre hypertrophy and increased SC abundance,^81^ suggesting that macrophages contribute to exercise‐induced muscle adaptation. Macrophages regulate largely via cytokines; for instance, macrophages release a metalloproteinase that induces SC activation by inhibiting Notch signalling.^S8^ Macrophages also release the cytokines IGF‐1, tumour necrosis factor alpha, IL‐1β, and interferon gamma.^82^ However, whether the above macrophage‐derived cytokines contribute to exercise‐induced muscle adaptation remains unknown.

The chemokine family consists of ~50 endogenous chemokine ligands in humans that play critical roles in haematopoiesis and immune cell homeostasis by controlling cell migration and cell positioning.^83^ Recent evidence has revealed the crucial functions of several chemokines in SC function.^84^ Macrophages secrete CXCL10, which induces the proliferation and expansion of SCs via its receptor CXCR3.^53^ Macrophages also secrete the cytokine nicotinamide phosphoribosyltransferase (Nampt), which promotes proliferation via the C‐C motif chemokine receptor type (CCR5) in SCs.^54^ Supplying Nampt had a positive effect on muscle regeneration,^54^ while CXCL10 treatment resulted in an 80% increase in the number of proliferative SCs and a 27% increase in the average myofibre CSA of regenerated myofibres in aged mice.^53^ In addition to blood cells, many chemokines and their receptors are expressed in mouse primary muscle cells.^84^ Moreover, an *in vitro* exercise model revealed that exercise induces the expression of multiple chemokines (e.g., CXCL1, CXCL12, CXCL5, CCL1, CCL2, and CCL7).^S43^ A four‐week period of running exercise induces CXCL12 and CXCR4 in MyHC‐positive muscle fibres, and the CXCL12‐CXCR4 axis promotes the differentiation of C2C12,^55^ suggesting the critical role of CXCL12 in exercise‐induced muscle hypertrophy. Chemokines are potentially useful for muscle maintenance in response to exercise and several chemokines are potential drug therapies; however, a more comprehensive view of exercise‐induced chemokines and their roles in SC function requires further investigation.

#### Hepatokines

Hepatokines are cytokines secreted by hepatocytes, and many hepatokines play substantial roles in crosstalk with muscle cells.^85^ For instance, the hepatokine apolipoprotein J (ApoJ) targets muscle glucose metabolism and insulin sensitivity.^86^ Moreover, hepatokines have been identified as molecular transducers of exercise and exercise induces the release of hepatokines, including FGF21, fetuin‐A, ANGPTL4, and follistatin.^11^, ^87^ Exercise alleviates cardiac fibrosis through increased FGF21, which regulates TGF‐β‐1‐Smad2/3‐MMP2/9 signalling in mice with myocardial infarction.^88^ FGF21 seems to regulate the differentiation of SCs; nevertheless, the exact role of FGF21 in SC function has not been determined. Interestingly, FGF19, a closely related endocrine FGF member, ameliorated muscle atrophy and alleviated sarcopenia,^89^ showing therapeutic potential for muscle wasting. Follistatin is a well‐known inducer of SC activation, and follistatin overexpression results in a 37% increase in muscle weight in animals by promoting SC proliferation through the inhibition of the expression of myostatin and activin.^S6^ Direct follistatin delivery preserved the SC pool after 6 months following reinnervation.^14^ The level of hepatokine selenoprotein P (SeP) seems not to be regulated by exercise in humans in response to acute moderate‐intensity exercise,^90^ but it increases with type 2 diabetes and aging.^91^ Inspiringly, SeP deficiency increased the responsiveness of skeletal muscle in mice and ameliorated muscle atrophy induced by immobilization.^84^, ^91^ SeP interacts with its muscle receptor low‐density lipoprotein receptor‐related protein (LRP1) and restricts the ROS production, AMPK phosphorylation, and PGC‐1α expression in skeletal muscle, suggesting that SeP inhibitors may act as exercise mimetics to treat muscle loss associated with a sedentary lifestyle or aging.^92^

#### Adipokines and neurokines

Adipokines are signalling molecules produced by adipose tissue (adipocytes are categorized as white, brown, or beige adipocytes).^93^ The interplay of adipose tissue and skeletal muscle is critical for metabolic homeostasis, as well as the important roles of several adipokines in SC function.^93^, ^94^ Loss of the adipokine lipocalin‐2 impaired SC activation and subsequent muscle regeneration by regulating the ECM protease matrix metalloproteinase‐9 (MMP‐9).^95^ Importantly, adiponectin levels increased after short‐term intense exercise (<60 min) in trained athletes.^96^ Globular adiponectin activates the small GTPase Rac1 to induce SCs to exit quiescence, which induces myogenesis in SCs upon muscle injury.^56^ Moreover, adiponectin deficiency results in perturbation of myogenesis in adiponectin knockout mice, suggesting the important role of adiponectin in muscle remodelling.^97^ Leptin is another adipokine in response to exercise, and long‐term exercise (≥60 min) reduces leptin release.^96^ Mice with a recessive mutation in the leptin receptor gene exhibited defective muscle regeneration, which was attributed to the perturbation of cell cycle reentry of SCs.^57^, ^58^ Muscle‐brain crosstalk has been confirmed, especially for exercise‐induced myokines.^98^, ^99^ Myokines, including FNDC5, cathepsin B, and IL‐6 mediate the exercise‐induced beneficial impact on neurogenesis.^99^ Brain‐derived neurotrophic factor (BDNF) is secreted mainly by the brain,^59^ and exercise has been shown to also induce the release of BDNF from skeletal muscle.^11^ Brain‐derived BDNF was essential for SC differentiation and muscle regeneration because BDNF knockout mice presented a decrease in the number of Pax7^+^ SCs and increased proliferation and differentiation of SCs via an uncovered mechanism.^60^, ^100^

#### Other exercise‐induced cytokines

Igfbp7 has been reported to be another myokine that promotes the self‐renewal of SCs.^15^ Exercise induces the expression of Igfbp7 in SCs, which inhibits Akt–mTOR signalling to protect SCs against exhaustion stress.^15^ The expression of FAM3A, a cytokine‐like protein, has been shown to increase in the livers of rats subjected to chronic and acute exercise.^101^ Further research demonstrated that myogenic cells also secreted FAM3A, which is required for SC commitment and muscle development via the promotion of oxidative metabolism.^102^ Voluntary exercise induces the release of vascular endothelial growth factor (VEGF) from skeletal muscle, which promotes angiogenesis in skeletal muscle.^103^ SCs recruit capillary endothelial cells (ECs) via VEGFA, and in return, ECs maintain SC quiescence though the EC‐derived Notch ligand Dll4.^104^ Leukaemia inhibitory factor (LIF) is also a myokine induced by exercise,^105^ It has been demonstrated that LIF‐treated SCs exhibit increased self‐renewal with upregulated expression of Pax7, thus showing enhanced transplantation efficiency in mice with Duchenne muscular dystrophy.^106^ Exercise augments the expression of granulocyte colony‐stimulating factor (G‐CSF) in TA muscles, suggesting that G‐CSF is an exercise‐induced myokine.^48^ Interestingly, G‐CSF replenished Pax7^high^ cells in aged mice by promoting the asymmetric division of Pax7^medium^ cells.^48^

In addition to the above findings, recent evidence revealed several exercise‐induced cytokines released by other types of cells that control SC homeostasis. Meterorin‐like hormone (Metrnl) is a novel myokine induced by exercise,^107^ while recent findings revealed that macrophage‐specific Metrnl depletion in mice severely impaired muscle regeneration.^S7^ At the molecular level, Metrnl induces Stat3 activation in macrophages, resulting in their differentiation to an anti‐inflammatory phenotype. Moreover, Metrnl induces the production of macrophage‐dependent insulin‐like growth factor, which directly promotes SC proliferation.^S7^ Erythropoietin (EPO) is secreted mainly by the kidney and liver, as well as by skeletal muscle.^108^ It has been revealed that 65 min of cycle exercise‐induced skeletal muscle releases EPO,^109^ suggesting that EPO is an exercise‐induced cytokine. Importantly, EPO treatment significantly enhanced muscle strength, as indicated by an increase in the number of BrdU‐positive SCs.^13^ Another finding showed that EPO promoted the expression of MyoD in SCs and inhibited the apoptosis of SCs.^61^ Tissue inhibitor of metalloproteinases 3 (TIMP3), a secreted metalloproteinase inhibitor released by fibro/adipogenic progenitors (FAPs) restrained intramuscular fat formation. A pharmacological mimetic of TIMP3 blocked the conversion of FAPs into adipocytes, suggesting a strategy to improve muscle regeneration.^110^

### The application of exercise‐induced cytokines in muscle maintenance

It is widely recognized that regular physical exercise greatly benefits muscle performance.^111^ The identification of molecules that mediate the beneficial effects of exercise has preventive and therapeutic implications in medicine. PGC‐1 coactivators regulate broad transcriptional programmes in response to exercise.^111^, ^112^ PGC‐1α muscle‐specific overexpression mice (MCK‐PGC‐1α) exhibit many adaptations to endurance training and resistance to muscle atrophy upon unloading, disuse, or denervation.^113^, ^114^ MCK‐PGC‐1α mice have been widely used as a model of endurance exercise, as their muscles are formed almost exclusively by oxidative myofibre.^113^, ^114^ PGC‐1α expression in myofibres favours pro‐myogenic and anti‐adipogenic cell populations in skeletal muscle.^115^ Moreover, PGC‐1α overexpression induces levels of fibronectin to remodel the SC niche, resulting in a greater propensity for activation and proliferation of SCs.^116^ Importantly, PGC‐1α has a profound effect on myokine expression.^117^ For instance, PGC‐1α overexpression promoted the secretion of B‐type natriuretic peptide (BNP), which activated tissue‐resident macrophages.^118^ Neurturin is also a PGC‐1a‐controlled myokine, and Neurturin‐transgenic mice largely recapitulated the phenotype observed in MCK‐PGC‐1α mice (improved exercise performance),^119^ implying the outstanding effect of exercise‐induced cytokines on muscle performance.

Indeed, the administration of selective exercise‐induced cytokines substantially enhanced muscle performance. In a mouse model of skeletal muscle injury, irisin injection (2.5 μg/g of body weight, three times a week) increased the percentage of regenerated myofibres containing two centrally formed nuclei (~30% to ~40%) and resulted in regenerative myofibres with large (>1500 μm^2^) CSA in mouse skeletal muscle.^12^ Moreover, irisin injection rescued denervation‐induced atrophy in mice, as shown by ~30% increase in the CSA of the tibialis anterior (TA).^12^ Irisin reduced mRNA and protein levels in muscle tissue during aging, while intraperitoneal administration of irisin (2 mg/kg body weight, three times per week) in aging (14‐month‐old) or aged (22‐month‐old) mice significantly improved sarcopenia, as indicated by increased strength (+18.42% or +13.88%) and muscle weights (+9.02% or + 16.39%).^120^ The cytokine EPO also obviously promoted muscle regeneration in a rat model, and injection of EPO (5000 IU/kg body weight) significantly improved muscle strength, as indicated by 10–20% greater twitch and tetanic force values and increased numbers of BrdU‐positive SCs.^13^ For aged‐related muscle function decline, recombinant CXCL10 treatment (1 μg per TA, at 1 and 3 days after injury) rejuvenated the number of Pax7^+^Ki67^+^ SCs and resulted in a 27% increase in the CSA of regenerated myofibres.^53^ A single dose of human recombinant Nampt (0.5 μg) at the time of injury increased the number of proliferating Pax7^+^ SCs and a 3.276 ± 0.4926 mm^2^ increase in muscle area.^54^ In a model of C26 colon carcinoma‐bearing mice, the administration of IL‐4 (1.3 μg per mouse, every day) improved cachexia, as shown by increased muscle mass, fibre CSA, and SC pool.^S39^ Moreover, in a mouse muscle atrophy model, the combined administration of HGF and LIF6 significantly increased the weight of the TA (9%), extensor digitorum longus (EDL) (18%), and CSA of EDL (22%).^52^ These inspiring findings demonstrate that several exercise‐induced cytokines can be applied as new therapeutic modalities for skeletal muscle injury and disease but still require further research.

## Conclusions and future perspectives

**Figure 1:**
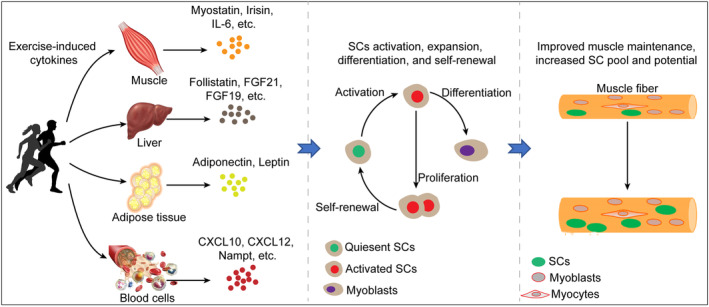
Overview of the exercise‐induced cytokines regulating SCs. Exercise induces the release of cytokines, including myokines, hepatokines, adipokines, from multiple tissues. These cytokines synergistically induce the activation, proliferation, and differentiation of SCs, with enhanced self‐renewal to preserve the SC pool. Specific exercise‐induced cytokines are critical mediators of exercise in muscle hypertension and have high therapeutic potential against muscle loss.

In this review, we summarize the identification of exercise‐induced cytokines and their roles in SCs. Overall, many exercise‐induced cytokines have been identified to play important roles in muscle maintenance and regeneration, as well as regulating the proliferation, differentiation, survival, and self‐renewal of SCs (Figure 1), suggesting that these cytokines may be useful for treating muscle wasting diseases. Despite the high therapeutic potential of exercise‐induced cytokines, several questions and directions should be considered. First, exercise‐induced cytokines still need to be completely identified and illustrated. Second, the exact effects and underlying mechanisms of these cytokines on SCs require further clarification. Third, attention should be given to developing efficient strategies to govern SC function and intervene in muscle loss, using exercise‐induced cytokines (alone or cocktail).

## Funding

This work was supported by grants from the National Natural Science Foundation of China (12272070) and the International Cooperation Project of China Manned Space Program.

## Conflict of interest

The authors declare that they have no competing interests.

## Supporting information


**Data S1.** Supporting Information.
