# Supplementary material for: Control of muscle satellite cell function by specific exercise‐induced cytokines and their applications in muscle maintenance
Source: J Cachexia Sarcopenia Muscle. 2024 Feb 20;15(2):466–76. doi: 10.1002/jcsm.13440 (PMC10995279; doi:10.1002/jcsm.13440)
Supplement: Supplementary file 1 — Data S1. Supporting Information. [file JCSM-15-466-s001.docx]

S1. Smith PJ, Merwin RM. The role of exercise in management of mental health disorders: an integrative review. *Annu Rev Med*. 2021;72:45-62.

**S2. Spaulding HR, Yan Z. AMPK and the adaptation to Exercise. *Annu Rev Physiol*. 2022;84:209-227.**

**S3. Herzig S, Shaw RJ. AMPK: guardian of metabolism and mitochondrial homeostasis. *Nat Rev Mol Cell Biol*. 2018;19(2):121-135.**

**S4. Bueno Júnior CR, Pantaleão LC, Voltarelli VA, Bozi LH, Brum PC, Zatz M. Combined effect of AMPK/PPAR agonists and exercise training in mdx mice functional performance. *PLoS One*. 2012;7(9):e45699.**

**S5. Fu X, Zhu M, Zhang S, Foretz M, Viollet B, Du M. Obesity impairs skeletal muscle regeneration through inhibition of AMPK. *Diabetes*. 2016;65(1):188-200.**

S6. Gilson H, Schakman O, Kalista S, Lause P, Tsuchida K, Thissen JP. Follistatin induces muscle hypertrophy through satellite cell proliferation and inhibition of both myostatin and activin. *Am J Physiol Endocrinol Metab*. 2009;297(1):E157-E164.

**S7. Baht GS, Bareja A, Lee DE, et al. Meteorin-like facilitates skeletal muscle repair through a Stat3/IGF-1 mechanism. *Nat Metab*. 2020;2(3):278-289.**

**S8. Du H, Shih CH, Wosczyna MN, et al. Macrophage-released ADAMTS1 promotes muscle stem cell activation. *Nat Commun*. 2017;8(1):669.**

**S9. Shang M, Cappellesso F, Amorim R, et al. Macrophage-derived glutamine boosts satellite cells and muscle regeneration. *Nature*. 2020;587(7835):626-631.**

S10. Zhang BT, Yeung SS, Liu Y, et al. The effects of low frequency electrical stimulation on satellite cell activity in rat skeletal muscle during hindlimb suspension. *BMC Cell Biol*. 2010;11:87.

S11. Joanisse S, Lim C, McKendry J, Mcleod JC, Stokes T, Phillips SM. Recent advances in understanding resistance exercise training-induced skeletal muscle hypertrophy in humans. *F1000Res*. 2020;9:F1000 Faculty Rev-141.

**S12. Wozniak AC, Anderson JE. Nitric oxide-dependence of satellite stem cell activation and quiescence on normal skeletal muscle fibers. *Dev Dyn*. 2007;236(1):240-250.**

S13. Joanisse S, Snijders T, Nederveen JP, Parise G. The impact of aerobic exercise on the muscle stem cell response. *Exerc Sport Sci Rev*. 2018;46(3):180-187.

S14. Brooks MJ, Hajira A, Mohamed JS, Alway SE. Voluntary wheel running increases satellite cell abundance and improves recovery from disuse in gastrocnemius muscles from mice. *J Appl Physiol (1985)*. 2018;124(6):1616-1628.

S15. Verney J, Kadi F, Charifi N, et al. Effects of combined lower body endurance and upper body resistance training on the satellite cell pool in elderly subjects. *Muscle Nerve*. 2008;38(3):1147-1154.

**S16. Flück M, Hoppeler H. Molecular basis of skeletal muscle plasticity--from gene to form and function. *Rev Physiol Biochem Pharmacol*. 2003;146:159-216.**

**S17. Mahoney DJ, Tarnopolsky MA. Understanding skeletal muscle adaptation to exercise training in humans: contributions from microarray studies. *Phys Med Rehabil Clin N Am*. 2005;16(4):859-vii.**

**S18. Mahoney DJ, Parise G, Melov S, Safdar A, Tarnopolsky MA. Analysis of global mRNA expression in human skeletal muscle during recovery from endurance exercise. *FASEB J*. 2005;19(11):1498-1500.**

**S19. Joanisse S, Nederveen JP, Baker JM, Snijders T, Iacono C, Parise G. Exercise conditioning in old mice improves skeletal muscle regeneration. *FASEB J*. 2016;30(9):3256-3268.**

**S20. McCarthy JJ, Mula J, Miyazaki M, et al. Effective fiber hypertrophy in satellite cell-depleted skeletal muscle. *Development*. 2011;138(17):3657-3666.**

**S21. Murach KA, White SH, Wen Y, et al. Differential requirement for satellite cells during overload-induced muscle hypertrophy in growing versus mature mice. *Skelet Muscle*. 2017;7(1):14.**

S22. Wen Y, Englund DA, Peck BD, Murach KA, McCarthy JJ, Peterson CA. Myonuclear transcriptional dynamics in response to exercise following satellite cell depletion. *iScience*. 2021;24(8):102838.

S23. Wei W, Riley NM, Yang AC, et al. Cell type-selective secretome profiling *in vivo*. *Nat Chem Biol*. 2021;17(3):326-334.

S24. Rundqvist H, Veliça P, Barbieri L, et al. Cytotoxic T-cells mediate exercise-induced reductions in tumor growth. *Elife*. 2020;9:e59996.

S25. Kerschan-Schindl K, Thalmann MM, Weiss E, et al. Changes in serum levels of myokines and wnt-antagonists after an ultramarathon race. *PLoS One*. 2015;10(7):e0132478.

S26. Lin J, Arnold HB, Della-Fera MA, Azain MJ, Hartzell DL, Baile CA. Myostatin knockout in mice increases myogenesis and decreases adipogenesis. *Biochem Biophys Res Commun*. 2002;291(3):701-706.

S27. Mitra A, Qaisar R, Bose B, Sudheer SP. The elusive role of myostatin signaling for muscle regeneration and maintenance of muscle and bone homeostasis. *Osteoporos Sarcopenia*. 2023;9(1):1-7.

S28. Budsuren U, Ulaangerel T, Shen Y, et al. MSTN regulatory network in mongolian horse muscle satellite cells revealed with miRNA interference technologies. *Genes (Basel)*. 2022;13(10):1836.

S29. Sheng H, Guo Y, Zhang L, et al. Proteomic studies on the mechanism of myostatin regulating cattle skeletal muscle development. *Front Genet*. 2021;12:752129.

S30. Rabiee F, Lachinani L, Ghaedi S, Nasr-Esfahani MH, Megraw TL, Ghaedi K. New insights into the cellular activities of Fndc5/Irisin and its signaling pathways. *Cell Biosci*. 2020;10:51.

S31. Roca-Rivada A, Castelao C, Senin LL, et al. FNDC5/irisin is not only a myokine but also an adipokine. *PLoS One*. 2013;8(4):e60563.

S32. Huh JY, Panagiotou G, Mougios V, et al. FNDC5 and irisin in humans: I. Predictors of circulating concentrations in serum and plasma and II. mRNA expression and circulating concentrations in response to weight loss and exercise. *Metabolism*. 2012;61(12):1725-1738.

S33. Missaglia S, Tommasini E, Vago P, et al. Salivary and serum irisin in healthy adults before and after exercise. *Eur J Transl Myol*. 2023;33(1):11093.

S34. Perakakis N, Triantafyllou GA, Fernández-Real JM, et al. Physiology and role of irisin in glucose homeostasis. *Nat Rev Endocrinol*. 2017;13(6):324-337.

S35. Boström P, Wu J, Jedrychowski MP, et al. A PGC1-α-dependent myokine that drives brown-fat-like development of white fat and thermogenesis. *Nature*. 2012;481(7382):463-468.

S36. Liang H, Qi W, Jiajue R, et al. Serum Irisin level is associated with fall risk, muscle strength, and cortical porosity in postmenopausal women. *Front Endocrinol (Lausanne)*. 2023;14:1096950.

S37. Wu J, Guan J, Lin S, et al. Prediction of sarcopenia among peritoneal dialysis patients using a combination of irisin and phase angle. *Nephrol Ther*. 2023;19(1):66-75.

**S38. Prokopchuk O, Liu Y, Wang L, Wirth K, Schmidtbleicher D, Steinacker JM. Skeletal muscle IL-4, IL-4Ralpha, IL-13 and IL-13Ralpha1 expression and response to strength training. *Exerc Immunol Rev*. 2007;13:67-75.**

**S39. Costamagna D, Duelen R, Penna F, Neumann D, Costelli P, Sampaolesi M. Interleukin-4 administration improves muscle function, adult myogenesis, and lifespan of colon carcinoma-bearing mice. *J Cachexia Sarcopenia Muscle*. 2020;11(3):783-801.**

S40. Broholm C, Mortensen OH, Nielsen S, et al. Exercise induces expression of leukaemia inhibitory factor in human skeletal muscle. *J Physiol*. 2008;586(8):2195-2201.

S41. Scheele C, Nielsen S, Pedersen BK. ROS and myokines promote muscle adaptation to exercise. *Trends Endocrinol Metab*. 2009;20(3):95-99.

S42. Kistner TM, Pedersen BK, Lieberman DE. Interleukin 6 as an energy allocator in muscle tissue. *Nat Metab*. 2022;4(2):170-179.

S43. Chen W, Nyasha MR, Koide M, et al. *In vitro* exercise model using contractile human and mouse hybrid myotubes. *Sci Rep*. 2019;9(1):11914.

**S44. Yasuda S, Goto Y, Takaki H, et al. Exercise-induced hepatocyte growth factor production in patients after acute myocardial infarction: its relationship to exercise capacity and brain natriuretic peptide levels. *Circ J*. 2004;68(4):304-307.**

**S45. Kolasa-Trela R, Konieczynska M, Bazanek M, Undas A. Specific changes in circulating cytokines and growth factors induced by exercise stress testing in asymptomatic aortic valve stenosis. *PLoS One*. 2017;12(3):e0173787.**

**S46. O'Reilly C, McKay B, Phillips S, Tarnopolsky M, Parise G. Hepatocyte growth factor (HGF) and the satellite cell response following muscle lengthening contractions in humans. *Muscle Nerve*. 2008;38(5):1434-1442.**
